# Supplementary material for: Enhancing lifespan of budding yeast by pharmacological lowering of amino acid pools
Source: Aging (Albany NY). 2021 Mar 21;13(6):7846–71. doi: 10.18632/aging.202849 (PMC8034917; doi:10.18632/aging.202849)
Supplement: Supplementary Figures [file aging-13-202849-s001.pdf]

SUPPLEMENTARY FIGURES

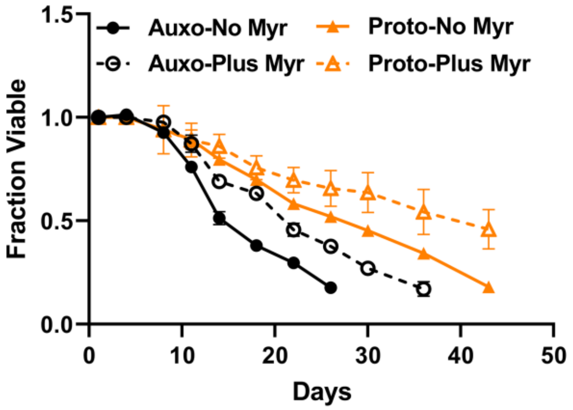

Supplementary Figure 1. Myr treatment increases longevity in buffered SDC medium. The lifespan of auxotrophic or prototrophic BY4741 cells was determined in SDC medium buffered with citrate-phosphate buffer and 1.50 µmol/L (600 ng/ml) of myriocin as previously described (Liu J, Huang X, Withers BR, Blalock E, Liu K, Dickson RC. Reducing Sphingolipid Synthesis Orchestrates Global Changes to Extend Yeast Lifespan. Aging Cell. 2013;12:833-41).

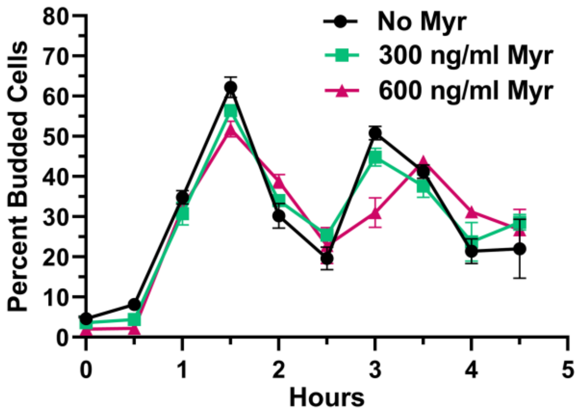

Supplementary Figure 2. Initial cell cycle progression. To examine how synchronized stationary phase cells are when inoculated into fresh SDC culture medium and to determine when they complete their first cell division cycle, we measured their budding behavior by using the Budding Index assay [1].

REFERENCES

1. Allen C, Büttner S, Aragon AD, Thomas JA, Meirelles O, Jaetao JE, Benn D, Ruby SW, Veenhuis M, Madeo F, Werner-Washburne M. Isolation of quiescent and nonquiescent cells from yeast stationary-phase cultures. J Cell Biol. 2006; 174:89–100. https://doi.org/10.1083/jcb.200604072 PMID:16818721

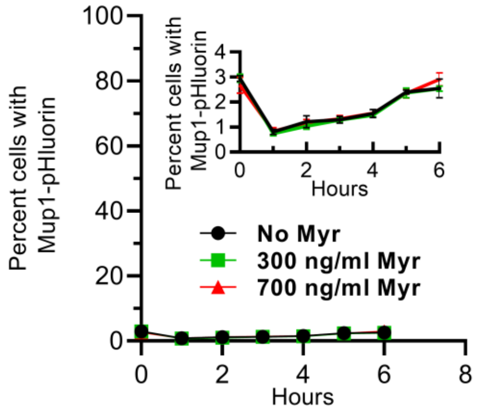

Supplementary Figure 3. Myr treatment reduces Mup1 activity in the PM. Flow cytometry analysis like that shown in Figure 5C, except cells were cultured in SDC medium, which contains 537 μmol/L methionine. As shown, flow cytometry results of cells cultured in this manner results in low surface expression of Mup1-pHluorin that is below the limit of useful detection for this assay. Thus, for culturing cells in SDC media, Mup1-FLAG was used since immunodetection of FLAG epitope in yeast lysates is more sensitive for measuring Mup1 expression (Figure 5E, 5F).
