# Supplementary material for: Enhancing lifespan of budding yeast by pharmacological lowering of amino acid pools
Source: Aging (Albany NY). 2021 Mar 21;13(6):7846–71. doi: 10.18632/aging.202849 (PMC8034917; doi:10.18632/aging.202849)
Supplement: Supplementary Table 1 [file aging-13-202849-s002.pdf]

SUPPLEMENTARY TABLE

Supplementary Table 1. Free amino acids pools.

Amino acid	No Myr (nmoles/1 x 10^7 cells)	Plus Myr (nmoles/1 x 10^7 cells)	p value	fold change
Ala	23.6	19.6	0.000299	0.83
Arg	51.1	27.8	2.03E-08	0.54
Asp	42.0	24.8	1.1E-07	0.59
Cys	2.6	1.5	0.109139	0.59
Glu	197.1	96.9	0.000108	0.49
Gly	22.4	11.7	3.03E-07	0.52
His	17.4	9.3	2.8E-08	0.53
Ile	5.6	3.2	3.18E-07	0.58
Leu	15.1	5.6	6.84E-09	0.37
Lys	64.0	20.7	4.41E-10	0.32
Met	7.2	0.8	2.07E-10	0.12
Phe	3.3	2.3	5.66E-05	0.70
Pro	7.1	5.0	0.00016	0.70
Ser	10.3	12.0	0.000552	1.16
Thr	21.9	22.5	0.189192	1.03
try	2.8	2.0	0.01478	0.71
val	36.8	9.5	3.75E-08	0.26

Free amino acid pools were measured in auxotrophic DBY747 cells (require Ura, Leu, Trp and His) grown in SDC medium buffered with succinate (Liu J, Huang X, Withers BR, Blalock E, Liu K, Dickson RC. Reducing Sphingolipid Synthesis Orchestrates Global Changes to Extend Yeast Lifespan. Aging Cell. 2013;12:833-41). Cells were grown from 0.005 to 2 A600nm units/ml at 30° C without and with 0.75 µmol/L (300 ng/ml) of myriocin. Amino acids were extracted by using the heat extraction procedure described in Materials and Methods and quantified by using a Hitachi L-8800A amino acid analyzer. The values for Asp are a combination of Asp and Asn, and for Glu they are combination of Glu and Gln. This occurs because the procedure for preparing samples for injection into the amino analyzer cause deamination of Asn and Gln. Both of these amino acids have small intracellular pools compared to Asp and Glu (see Figure 2). AUC Auxo-No Myr vs Auxo-Plus Myr (95% CI 14.89 to 15.97 vs 20.46 to 21.90). AUC Proto-No Myr vs Proto-Plus Myr (95% CI 25.54 - 26.66 vs 27.82 - 33.65).
